# Does Diffusely Infiltrating Lobular Carcinoma of the Breast Arise from Epithelial–Mesenchymal Hybrid Cells?

**DOI:** 10.3390/ijms241310752

**Published:** 2023-06-28

**Authors:** László Tabár, Renáta Bozó, Peter B. Dean, Katalin Ormándi, Olga Puchkova, Orsolya Oláh-Németh, István Balázs Németh, Zoltán Veréb, Ming-Fang Yen, Li-Sheng Chen, Hsiu-Hsi Chen, András Vörös

**Affiliations:** 1 Falun Central Hospital, Lasarettsvägen 10, 791 82 Falun, Sweden; 2 Department of Dermatology and Allergology, Albert Szent-Györgyi Medical School, University of Szeged, Korányi Street 6, H-6720 Szeged, Hungary; bozo.renata@med.u-szeged.hu (R.B.); nemethistvanbalazs@gmail.com (I.B.N.); vereb.zoltan@med.u-szeged.hu (Z.V.); 3 Department of Diagnostic Radiology, Faculty of Medicine, University of Turku, FI-20014 Turun, Finland; peter.dean@utu.fi; 4 Department of Radiology, University of Szeged, Semmelweis Street 6, H-6725 Szeged, Hungary; ormikati@gmail.com; 5 Department of Breast Imaging, Il’inskaya Hospital, Novorizhskoye Highway 9 km, 101000 Moscow, Russia; dachs@specialplan.net; 6 Department of Pathology, University of Szeged, Állomás Street 2, H-6725 Szeged, Hungary; orsolyaoolah@gmail.com (O.O.-N.); andrasvoros@libero.it (A.V.); 7 School of Oral Hygiene, College of Oral Medicine, Taipei Medical University, Wuxing Street, Taipei 110, Taiwan; amyyen@tmu.edu.tw (M.-F.Y.); samchen@tmu.edu.tw (L.-S.C.); 8 Institute of Epidemiology and Preventive Medicine, College of Public Health, National Taiwan University, 17 Hsuchow Road, Taipei 100, Taiwan; chenlin@ntu.edu.tw

**Keywords:** breast neoplasms, pathologists, interdisciplinary communication, mammography, patient care, histopathology technology, early detection of cancer, biomarkers, precision oncology, neoplastic stem cell

## Abstract

Classic diffusely infiltrating lobular carcinoma has imaging features divergent from the breast cancers originating from the terminal ductal lobular units and from the major lactiferous ducts. Although the term “invasive lobular carcinoma” implies a site of origin within the breast lobular epithelium, we were unable to find evidence supporting this assumption. Exceptional excess of fibrous connective tissue and the unique cell architecture combined with the aberrant features at breast imaging suggest that this breast malignancy has not originated from cells lining the breast ducts and lobules. The only remaining relevant component of the fibroglandular tissue is the mesenchyme. The cells freshly isolated and cultured from diffusely infiltrating lobular carcinoma cases contained epithelial–mesenchymal hybrid cells with both epithelial and mesenchymal properties. The radiologic and histopathologic features of the tumours and expression of the mesenchymal stem cell positive markers CD73, CD90, and CD105 all suggest development in the direction of mesenchymal transition. These hybrid cells have tumour-initiating potential and have been shown to have poor prognosis and resistance to therapy targeted for malignancies of breast epithelial origin. Our work emphasizes the need for new approaches to the diagnosis and therapy of this highly fatal breast cancer subtype.

## 1. Introduction

The initiation of nationwide mammography screening has brought about a paradigm shift in the size and stage of breast cancer at the time of diagnosis and treatment, from mainly palpable tumours to mainly nonpalpable tumours at an earlier phase of development [1]. Arresting the disease process has significantly reduced mortality from breast cancer in screened populations, a benefit restricted to women who have actually participated in screening [2,3]. Despite regular screening and the use of modern therapeutic regimens, the disease-specific outcome of some breast cancer subtypes has not been measurably improved [4,5].

To investigate why some breast cancers are still fatal despite our best efforts, we have used a prospectively collected database first established in 1977 as part of the Two-County Swedish Randomized Controlled Trial [6]. This database was expanded with data collected during the period of 1987–2019 from the ongoing service screening for all women aged 40–69 years in Dalarna County, Sweden (mean number, 52,438 women aged 40–69 years) [7]. The data included the mammographic features of the breast tumours (imaging biomarkers), which we found to possess valuable prognostic information [8,9]. The mammographic imaging biomarkers in correlation with large-format thin- and thick-section histopathology confirmed that the site of origin of breast cancer is an important determinant of outcome [4,8,10].

In our endeavour to find the sites of origin of breast cancer and link these with histological features and outcome, we have identified three major sites as follows: the major lactiferous ducts, the acini/terminal duct of the TDLU (terminal ductal lobular unit), and the fibrous connective tissue (mesenchyme) [11]. Ductal adenocarcinoma of the breast (DAB) originates from the epithelial cells lining the major lactiferous ducts. The malignant cells propagate within the existing ducts and also form new, pathologic, ductlike structures (neoductgenesis) [12,13,14,15,16,17]. In acinar adenocarcinoma of the breast (AAB), the cancer appears to originate from the epithelial cells of the acini and the terminal duct of the TDLU [18,19]. The AABs are of truly lobular origin while the diffusely infiltrating breast cancer, conventionally termed classic infiltrating lobular carcinoma of diffuse type [20], has entirely different clinical, imaging, and histopathologic characteristics compared to those cancers that originate from the major ducts and lobules, suggesting that it has a site of origin quite different from that of all other breast cancers. The site of origin of breast cancer should be an essential component of precision medicine, since as cancers originating in the acinar cells of the TDLU, the AABs are controllable by early detection through mammography screening and currently available therapy. On the contrary, the cancers originating in the major lactiferous ducts and the cancers originating presumably in the hybrid cells of the mesenchyme, the topic of this paper, are poorly controlled using current diagnostic and therapeutic methods [11].

**Figure 1:**
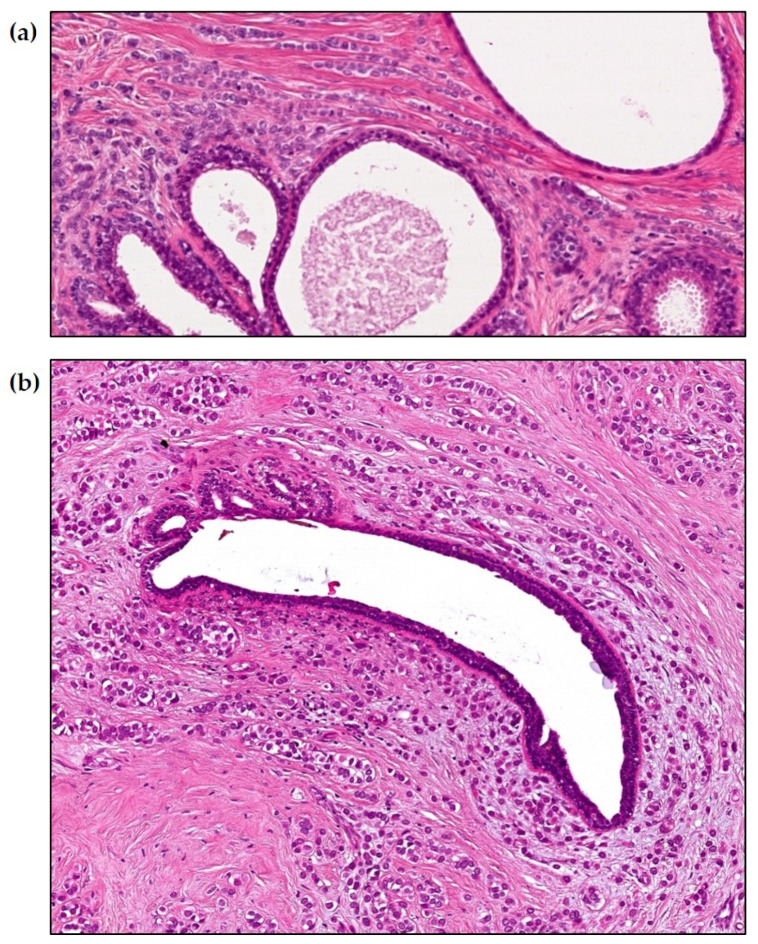
Details of large-format thin-section histopathology images of diffusely infiltrating lobular carcinoma. The invasive carcinoma surrounds the lobular acini (**a**) and a duct (**b**). The epithelial cells of both the lobular acini and the duct are normal, free of atypia, and free of evidence of lobular hyperplasia or LCIS (lobular cancer in situ), despite scrutiny using large-format sections (hematoxylin-eosin staining (H&E), 10× magnification).

In addition to the unique cell architecture, an excessive amount of fibrous connective tissue is the dominant feature of this malignancy, which characteristically has concave contours against the surrounding adipose tissue, as opposed to the convex contour of the adenocarcinomas originating from the lobule. This feature, along with an absence of malignant-type calcifications, makes it difficult to detect on the mammogram even if it is extensive at the time of detection. The unusual clinical, histopathologic, and imaging aspects of diffusely infiltrating breast cancer are not consistent with a site of origin in the cells lining breast ducts and lobules. Foote and Stewart originally described the “typical features” for invasive lobular carcinoma in 1946 as “Circumferential growth around non-neoplastic ducts (the targetoid pattern) and arrangement in a linear pattern (Indian files)” [21]. Figure 1 provides a histopathologic demonstration how “invasive lobular carcinoma” typically surrounds the acini and major ducts, which contain entirely normal cells, making it unlikely that the “invasive lobular carcinoma” could have arisen from the epithelial cells lining the acini of the lobule or the lactiferous ducts. In our search for the origin of this breast cancer subtype, we isolated and cultured cells derived from breast cancers having the characteristic clinical, imaging, and histopathologic features of “classic diffusely infiltrating lobular carcinoma”. This report describes the results of these cell cultures.

## 2. Results

### 2.1. Cultured Cells Had Long-Term Survival with Distinct Morphologic Phenotypes

**Figure 2:**
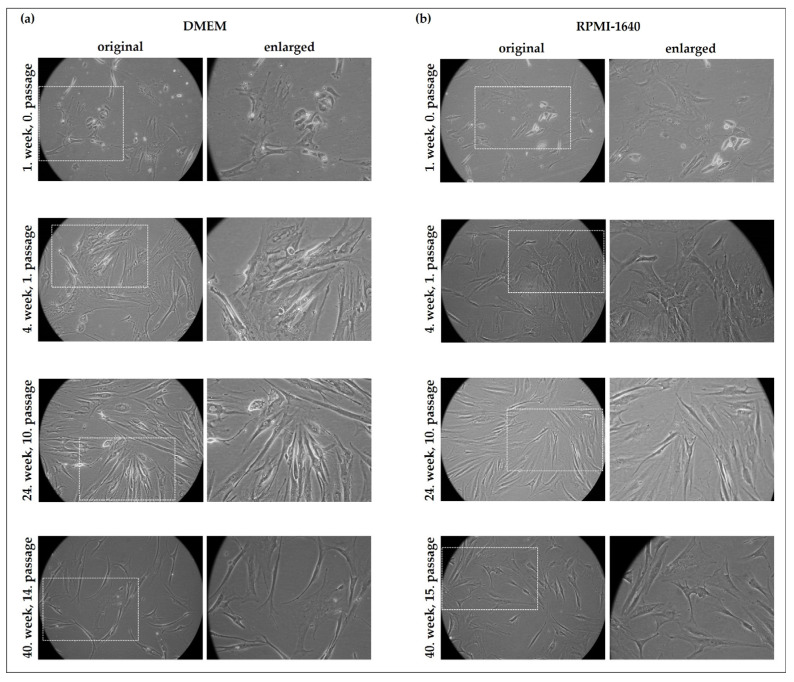
Cultured cells from diffusely infiltrating breast cancer. Long-term follow-up of the cells isolated from diffusely infiltrating breast cancer in Dulbecco’s Modified Eagle Medium (DMEM) with high glucose (**a**) and in RPMI-1640 medium (**b**). The cell growth was monitored by phase-contrast microscopy (20× original magnification). Dashed lines indicate the enlarged regions of the images.

We observed that the cultured cells survived for more than one year with continuous culturing and showed distinct morphologic phenotypes, which were sustainable during culturing in RPMI-1640 (+10% FBS) and in DMEM high-glucose culture media (+10% FBS) as well (Figure 2). RPMI-1640 (Roswell Park Memorial Institute) and DMEM media are widely used for culturing mammalian cells, including cancer cells and mesenchymal stem cells. Some of the cultured cells reached the 15th passage during this period, suggesting slow growth with long-term cell survival. These results also indicate a mixed population with fibroblastoid and mesenchymal stem-cell-like morphology.

### 2.2. Microtumour Formation by Cultured Cells

**Figure 3:**
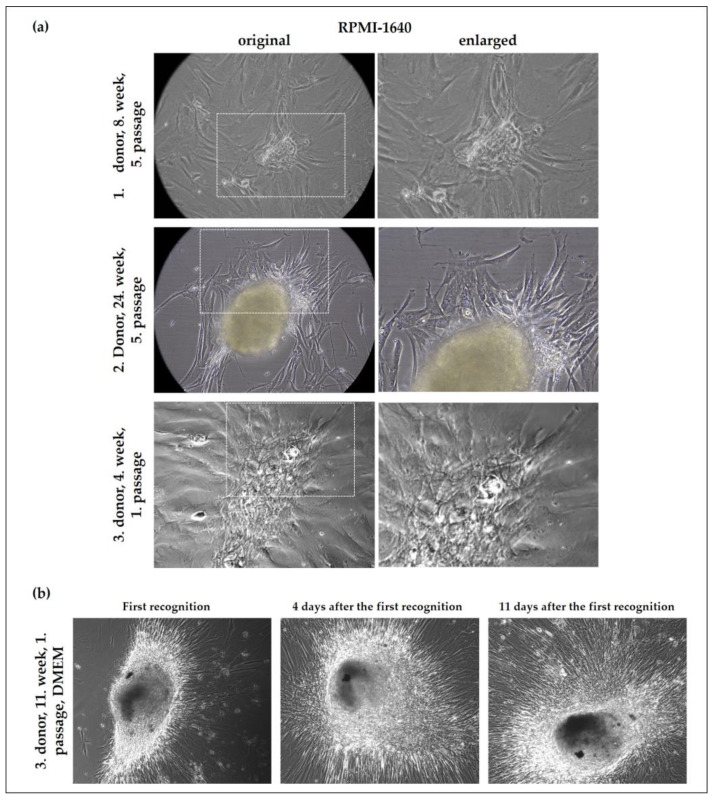
Microtumour-like phenotype of cells derived from diffusely infiltrating breast cancer showing the ability of the cultured cells to form microtumours regardless of time, culture media, and passage number. Cells were cultured in RPMI-1640 medium (20× original magnification) (**a**). Follow-up of a microtumour grown in Dulbecco’s Modified Eagle Medium (DMEM) (5× original magnification) (**b**).

During the long-term follow-up of the cell cultures isolated from these three separate cases of diffusely infiltrating breast cancer, we observed the formation of microtumours in the cell culture flasks from each of the three tumours. Interestingly, the pattern of the observed microtumours was similar to the tumour’s appearance on the mammogram. Microtumour formation was independent of the passage number, the culturing time, and the culture media (Figure 3), showing that we have isolated tumour cells which were capable of initiating tumour formation in a simple culture dish without any feeder-layer, additional immune cells, or extracellular matrix components.

### 2.3. Mesenchymal Stem Cell Marker Expression

**Figure 4:**
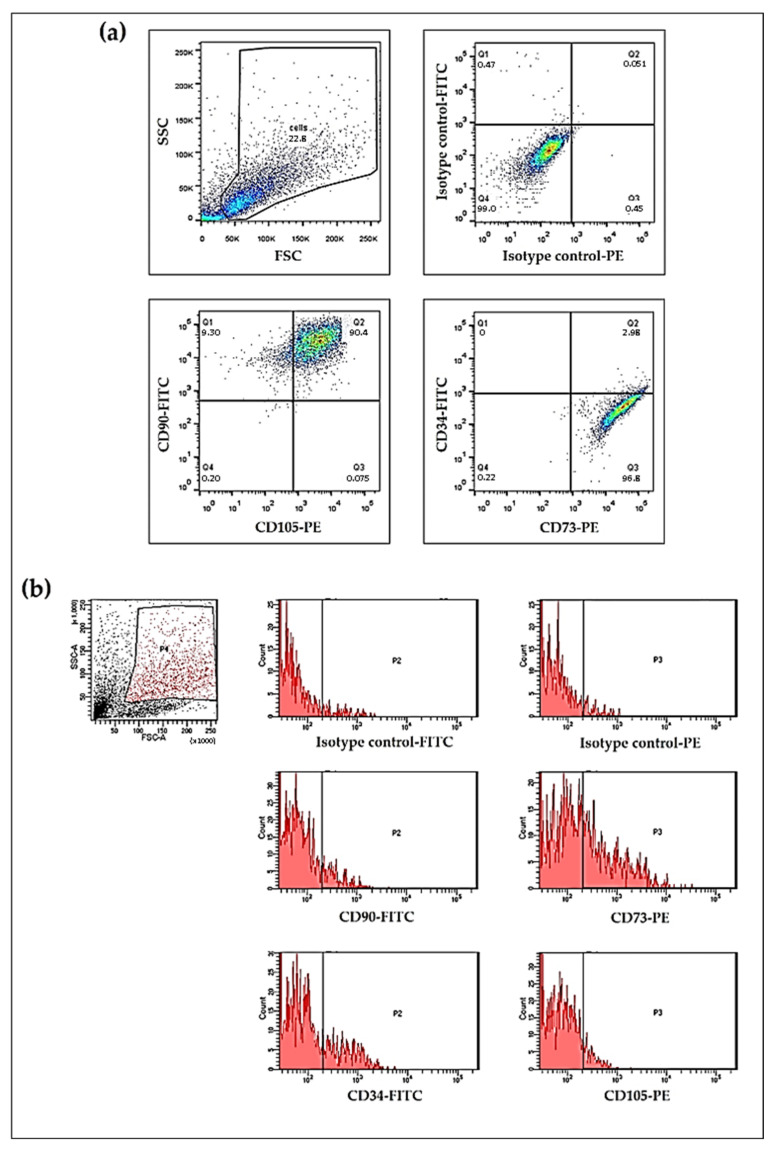
Flow cytometric analysis of the mesenchymal stem cell surface markers. Representative plots of the analysis of the mesenchymal stem cell related markers, CD34, CD73, CD90, and CD105 on the surfaces of cells originating from the three cases cultured in RPMI-1640 medium in the 4th passage (**a**) and on the surfaces of the freshly isolated cells (**b**).

We demonstrated that the cultured cells were epithelial–mesenchymal hybrid cells [22,23] that could form microtumours. These displayed both epithelial and mesenchymal features and had long-term survival with relatively low passage numbers. We examined the mesenchymal stem cell markers (positive markers: CD73, CD90, CD105; negative marker: CD34 [24]) on the cultured and freshly isolated cells to search for hybrid cells in the culture and tumour tissue which can potentially lead to the development of diffusely infiltrating carcinoma. The presence of CD73, CD90, and CD105 cell surface markers indicates their epithelial–mesenchymal hybrid cell nature. A large population of the examined cultured cells in RPMI-1640 medium in the 4th passage were CD90 and CD105 double positive. Using CD34 and CD73 double labelling, we observed that most of the cells were positive for CD73 and negative for CD34. These observations are presented in Figure 4a. Analysis of the freshly isolated cells showed that the larger, more granulated cells, which could be potential epithelial–mesenchymal hybrid cells, were also positive for CD90, CD105, and CD73. Although we also observed cells positive for CD34, all the investigated mesenchymal stem cell positive markers, including CD73, CD90, and CD105, were also detectable in the freshly isolated cell population. These observations are presented in Figure 4b. These results demonstrate the presence of mesenchymal stem cell marker positive cells in the tissue of the three diffusely infiltrating carcinoma cases and also in the cultured cells grown from these three separate tumours.

### 2.4. Epithelial–Mesenchymal Hybrid Cell Characteristics

**Figure 5:**
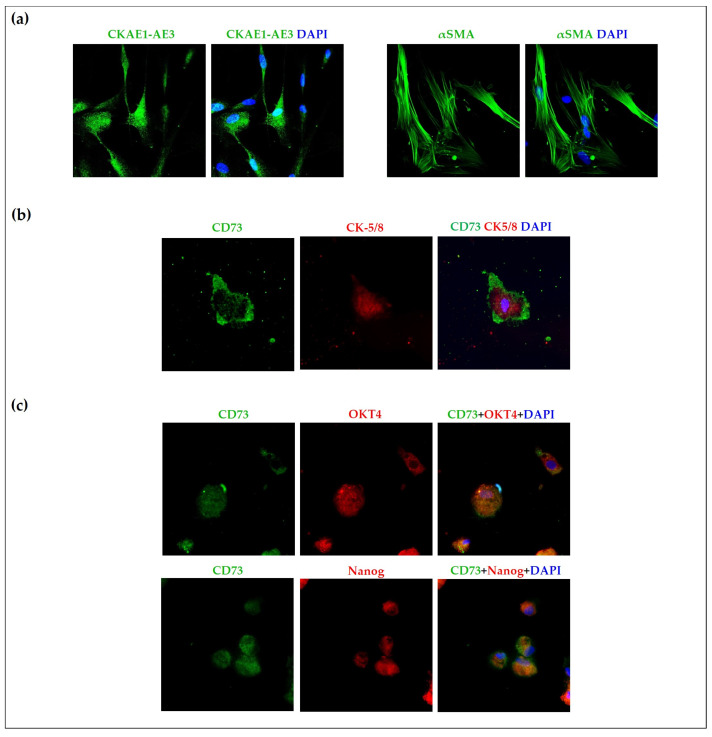
Mesenchymal-like characteristics of the cultured cells. Immunofluorescence staining for cytokeratin AE1-AE3 and smooth-muscle actin (**a**), CD73-cytokeratin 5/8 (**b**), and CD73-OKT4 and CD73-Nanog (**c**) in cells cultured from diffusely infiltrating breast cancer in RPMI-1640 medium (n = 3). Nuclei were visualized by 4′,6-diamidino-2-phenylindole (DAPI) staining (40× original magnification with immersion oil; Zeiss Axio Imager Z1 and Zeiss LSM 880 microscopes (Carl Zeiss AG, Oberkochen, Germany)).

Although both the cultured and freshly isolated cells expressed mesenchymal stem cell positive markers, we investigated further potential epithelial–mesenchymal hybrid cell characteristics of the cultured cells. Cytokeratin AE1-AE3 and α-SMA staining were used to distinguish the epithelial–mesenchymal hybrid cells in the culture from stromal cells such as fibroblasts. We found that freshly isolated and cultured cells in RPMI-1640 media in the 4th passage were positive for cytokeratin AE1-AE3 and α-SMA. These observations are presented in Figure 5a. Furthermore, the mesenchymal stem cell marker (CD73) was expressed by cells on cytospin preparations from the same cultures that also expressed cytokeratin 5/8. These observations are presented in Figure 5b. Since OKT4 and Nanog can play a role in the maintenance of the pluripotency of epithelial–mesenchymal hybrid cells, CD73-OKT4 and CD73-Nanog double immunofluorescence staining was also applied on cytospin preparations. These cultured cells were positive for OKT4 and Nanog as well, presented in Figure 5c, demonstrating that these cells have an epithelial–mesenchymal phenotype and are not stromal cells such as fibroblasts.

## 3. Discussion

Cells originating from classic, diffusely infiltrating lobular carcinoma survived for more than a year in cell culture, formed microtumours and extensive interaction net-works, and expressed pluripotency markers, all indicating their tumorigenic potential. The cells also showed fibroblastoid and epithelial–mesenchymal hybrid cell morphology in culture and expressed mesenchymal stem cell positive markers (CD70, CD90, and CD105). The epithelial–mesenchymal hybrid cell characteristics of the cultured cells were similar to the characteristics of the freshly isolated cells, indicating the presence of these hybrid cells in the original tumour tissue. Furthermore, the low proliferative capacity of the cultured cells is in line with the low expression of the Ki67 proliferation marker observed in all the investigated tumour tissues. Our results suggest that this malignancy contains epithelial–mesenchymal hybrid cells capable of initiating a tumour-forming process. In addition to the unusual clinical, imaging, and histopathologic aspects of diffusely infiltrating breast cancer, these cell culture findings also fail to support the current assumption that this breast cancer subtype has its origin in the epithelial cells of the breast lobules.

The macroscopic structure of this breast cancer subtype, as depicted by mammography, ultrasound, magnetic resonance imaging, and subgross, large-format, thick-section (3D) histopathology, closely resembles the structural framework (mesenchyme) of the normal breast until the massive accumulation of newly formed fibrous tissue distorts the architectural integrity at a late phase of tumour development. The resulting imaging biomarker, architectural distortion, eventually enables detection by breast imaging, and the massive collagen accumulation enables detection by palpation. The proliferation of mesenchymal elements indicates a possible mesenchymal origin, where both epithelial and mesenchymal transition may coexist. Epithelial–mesenchymal hybrid cells have been shown to have poor prognosis, tumour-initiating potential, and resistance to therapy targeted for malignancies of breast epithelial origin. Unfortunately, therapy is currently selected under the belief that this malignancy has originated from the epithelial cells of the breast lobules. This belief may prevent patients from receiving appropriately targeted therapy.

So long as this unusual breast malignancy is termed “classic diffusely infiltrating lobular carcinoma”, implying that it has its origin in the terminal ductal lobular units (TDLUs) of the breast, we are unlikely to achieve any real progress in our efforts to control it. A terminology which specifically indicates its actual origin will be a necessary first step in evaluating new approaches to detection and treatment [11]. Proof of an epithelial–mesenchymal hybrid cell origin could open the way for the development of new therapeutic approaches to breast cancer management aimed at better control of this complex breast cancer subtype. While this subtype accounts for approximately 5–8% of all breast cancers, it accounts for a disproportionately large percentage of all breast cancer deaths.

## 4. Materials and Methods

Three cases were selected for this study after obtaining written informed consent from the patients and approval from the Human Investigational Review Board, in accordance with the rules of the Helsinki Declaration. None of the patients had been subjected to preoperative chemotherapy or radiation therapy. The study was approved by the Human Investigation Review Board of the University of Szeged, Hungary, permission number: 5052/21-SZTE.

### 4.1. Detailed Description of Case 1

**Figure 6:**
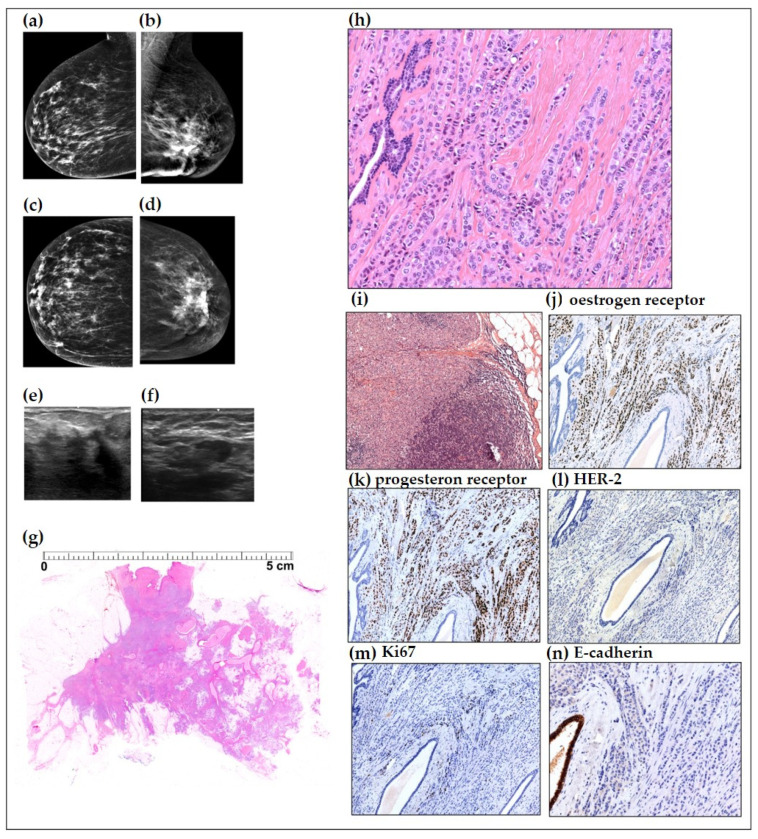
Case 1. Imaging and histopathological findings. Mediolateral (**a**,**b**) and craniocaudal (**c**,**d**) mammograms showing a normal right breast and the left breast grossly deformed by an extensive malignant tumour having architectural distortion and no associated calcifications. Handheld ultrasound: a large anechoic malignant tumour with pathologic axillary lymph nodes (**e**,**f**). Low-power, large-format histopathology image of a portion of this histologically 102 mm moderately differentiated, diffusely infiltrating carcinoma (**g**) (H&E staining). Higher-power histopathology images show a classical single-file growth pattern characteristic for “classic infiltrating lobular carcinoma” (**h**). Histopathology image of the axillary lymph node metastases (**i**). The immuno-histochemical staining showed strong diffuse oestrogen receptor (**j**) and progesterone receptor (**k**) positivity in 95% of the tumour cells, human epidermal growth factor receptor 2 (HER-2) negativity (**l**), 15% proliferation index (Ki 67) (**m**), and the absence of E-cadherin (**n**). 10× magnification: (**h**,**i**,**k**–**n**); 5× magnification: (**j**).

**Figure A1:**
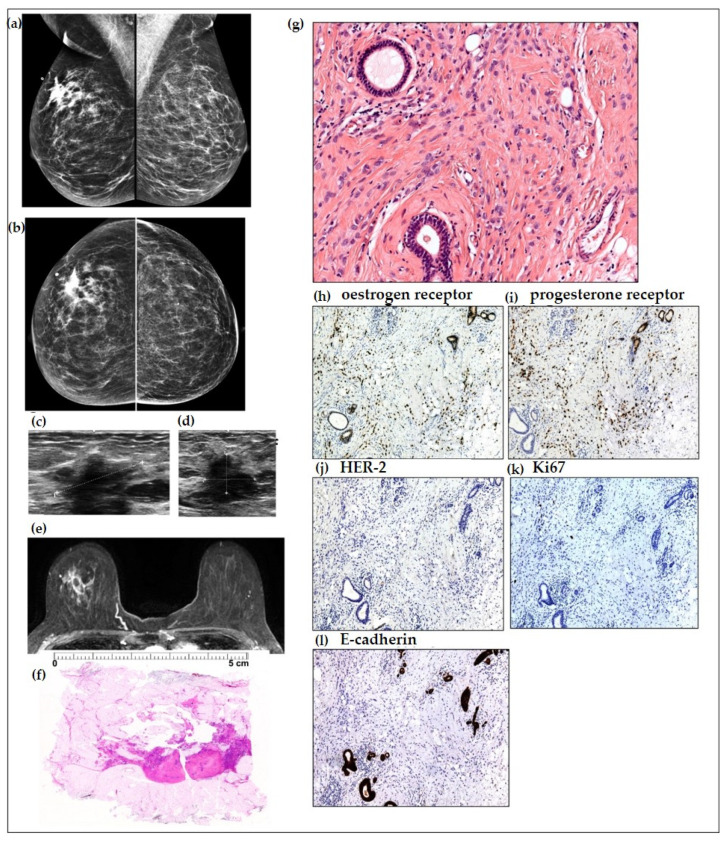
Case 2. Imaging and histopathological findings. Mediolateral (**a**) and craniocaudal (**b**) mammograms showing a normal left breast and an extensive region of architectural distortion in the upper-outer quadrant of the right breast with no associated calcifications. Handheld ultrasound shows an anechoic malignant tumour (**c**,**d**). Magnetic resonance imaging demonstrates the architectural distortion of the enhancing tumour (**e**). Low-power, large-format histopathology image of this 42 mm moderately differentiated, diffusely infiltrating carcinoma (**f**). Higher-power histopathology image shows features of a “classic infiltrating lobular carcinoma” (**g**). No lymphovascular, perivascular, or perineural invasions were demonstrable. The immunohistochemical staining showed strong diffuse oestrogen receptor (**h**) and progesterone receptor (**i**) positivity in 95% of the tumour cells, human epidermal growth factor receptor 2 (HER-2) negativity (**j**), 10% proliferation index (Ki 67) (**k**), and the absence of E-cadherin (**l**). (magnification: 10×).

**Figure A2:**
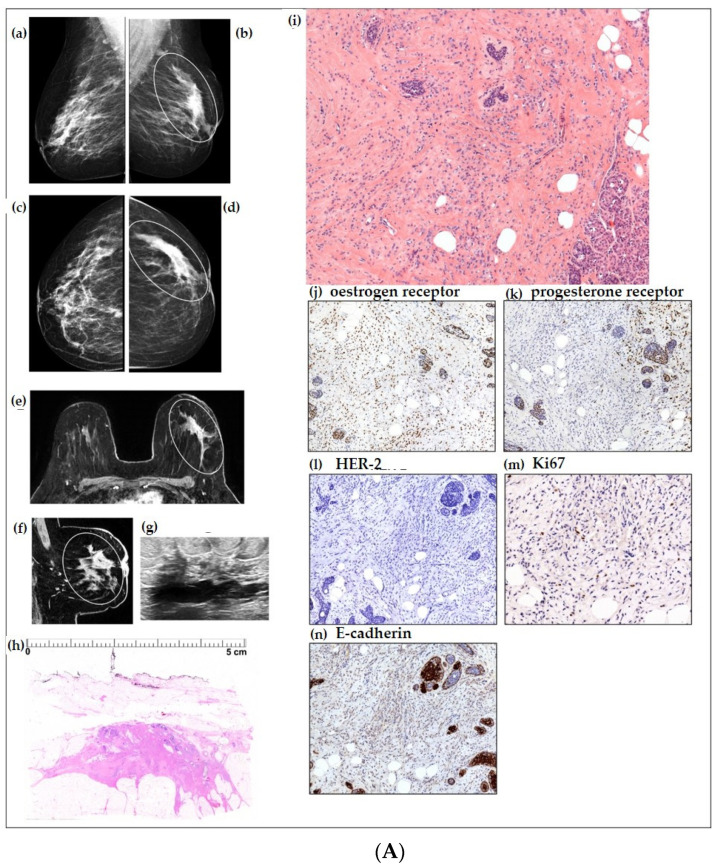

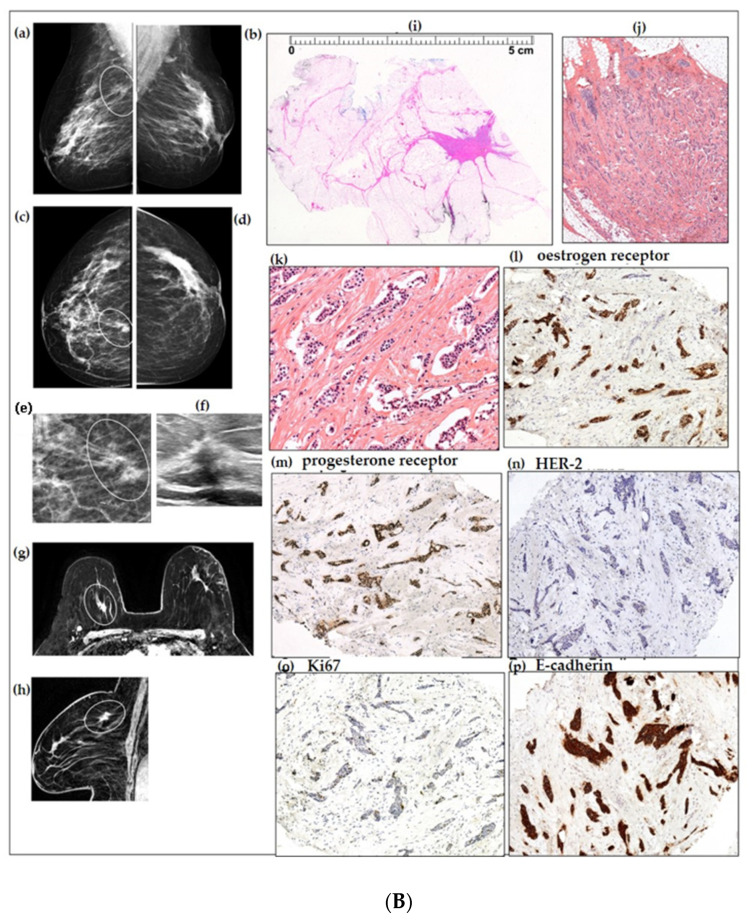
(**A**) Case 3. Imaging and histopathological findings in the left breast. Mediolateral (**a**,**b**) and craniocaudal (**c**,**d**) mammograms. Corresponding to the palpable tumour in the upper-outer quadrant of the left breast, there is a diffuse, non-specific asymmetric density with no associated calcifications, extending from the retracted nipple to the chest wall (**b**,**d**). There is a mammographically detected, 20 mm stellate tumour mass in the upper-inner quadrant of the right breast (**a**,**c**) (see description in (**B**)). Magnetic resonance images (**e**,**f**) demonstrate the extensive non-mass enhancement in the left breast and the small stellate tumour mass in the right breast (see description in (**B**)). Handheld ultrasound shows a large anechoic malignant tumour in the left breast (**g**). Low-power, large-format histopathology image (**h**) of a portion of this histologically 75 mm moderately differentiated, diffusely infiltrating carcinoma. The histopathologic features of a “classic infiltrating lobular carcinoma” are shown on the higher-power images of the left breast specimen (**i**). The immunohistochemical staining showed strong diffuse oestrogen receptor (**j**) and progesterone receptor (**k**) positivity in 95% of the tumour cells, human epidermal growth factor receptor 2 (HER-2) negativity (**l**), 5% proliferation index (Ki 67) (**m**), and the absence of E-cadherin (**n**). (**B**) Case 3. Imaging and histopathological findings in the right breast. Mediolateral (**a**,**b**) and craniocaudal (**c**,**d**) mammograms. There is a mammographically detected, 18 mm stellate tumour mass in the upper-inner quadrant of the right breast (**a**,**c**). Microfocus magnification of the mammographically malignant stellate tumour (**e**). Handheld ultrasound of the right breast confirms the malignant appearance of this small stellate tumour (**f**). Magnetic resonance images of the right breast demonstrate a unifocal stellate tumour mass (**g**,**h**). Low-power, large-format histopathology image of this 18 mm invasive breast cancer (**i**). Intermediate (**j**) and high power (**k**) histopathology images of this moderately differentiated breast cancer, acinar adenocarcinoma of the breast, AAB. The immunohistochemical staining showed diffuse oestrogen receptor (**l**) and progesterone receptor (**m**) positivity in 90% of the tumour cells, human epidermal growth factor receptor 2 (HER-2) negativity (**n**), 5% proliferation index (Ki 67) (**o**), and strongly positive E-cadherin staining (**p**). (magnification: 10×).

A 68-year-old woman presented with a self-detected tumour in her left breast. Clinical breast examination confirmed the presence of a large, firm, clearly malignant tumour deforming the lower half of the left breast. Mammographic, ultrasound, and histopathologic images are shown on Figure 6. Eleven months following mastectomy, the patient was diagnosed with peritoneal carcinomatosis and extensive bone metastases, and she died one year following mastectomy. A detailed description of Cases 2 and 3 is presented in the Appendix A, Figure A1 and Figure A2.

### 4.2. Cell Isolation from Tumour Tissue

The selected tumour tissue was cut into small pieces with a razor blade in a Petri dish and washed with Salsol solution containing 1% antibiotic/antimycotic solution (AB/AM). Subsequently, cells were isolated using an enzymatic-based method. Briefly, samples were digested in dispase neutral protease, grade II (2 U/mL) solution for 60 min at 37 °C. Following this, the tissue pieces were placed into a Dulbecco’s Modified Eagle Medium (DMEM) containing 1 g/litre glucose, collagenase from Clostridium histolyticum (2.7 mg/mL), deoxyribonuclease I from bovine pancreas (0.1 mg/mL), hyaluronidase from bovine testes (1.25 mg/mL) and foetal bovine serum (FBS, 2.5%) and incubated for 60 min at 37 °C. After the digestion, 5 mL phosphate-buffered saline–ethylenediaminetetraacetic acid (PBS-EDTA) was added and the samples were suspended continuously for 5 min. The suspensions were filtered into a centrifuge tube using 100 μm filter and centrifuged in the presence of FBS (1150 rpm, 10 min, at 4 °C). For culturing the cells, two different culture media were used: (1) DMEM 4.5 g/L glucose supplemented with 10% FBS, 1% (AB/AM), and 1% L-glutamine. (2) RPMI-1640 medium supplemented with 10% FBS, 1% AB/AM, and 1% L-glutamine. Cells were cultured at 37 °C in a humidified atmosphere with 5% CO_2_. The cell growth was monitored by phase-contrast microscope (Nikon eclipse TS100 (Nikon Corp. Minato City, Tokyo, Japan), or Zeiss Axiolab Vert.A1 microscopes(Carl Zeiss AG, Oberkochen, Germany)).

### 4.3. Flow Cytometric Analysis

For flow cytometric analysis of the potential mesenchymal stem cell characteristics, cells were harvested by short trypsinization and collected by centrifugation. Next, collected cells were washed with FACS buffer and incubated for 30 min with fluoro-chrome-conjugated antibodies: anti-human cluster of differentiation (CD) 34-FITC, CD73-PE, CD90-FITC, and CD105-PE, and anti-human IgG1-FITC/PE as an isotype control. Cells were measured by BD FACSAria Fusion flow cytometer (Becton, Dickinson and Company, Franklin Lakes, NJ, USA). Data were analysed using BD FACS Diva v6.0 and FlowJo software v7.6.5 (Becton, Dickinson and Company, Franklin Lakes, NJ, USA).

### 4.4. Immunofluorescence Staining

Cytospin preparations from freshly isolated cells and cultured cells (RPMI, 14th passage) from diffusely infiltrating breast cancer were made with Wescor Cytopro 7620 Cytocentrifuge at 600 rpm for 6 min and fixed in 4% paraformaldehyde (PFA). An amount of 0.25% Triton-X100 was applied for permeabilization, and 0.5% bovine serum albumin and normal goat serum in Tris-buffered saline were applied for blocking the non-specific sites. For specific labelling, the following primary antibodies were applied: mouse anti-human CD73, FITC-conjugated cytokeratin 5/8, rabbit anti-human OKT4, rabbit anti-human Nanog, and mouse anti-human CD105, with acetone fixation and permeabilization. Mouse anti-human cytokeratin AE1-AE3 and mouse anti-human α-smooth-muscle actin (α-SMA) antibodies were applied with the same protocol on cells which were grown on 8-well chamber slides. Alexa Fluor 647-conjugated anti-mouse IgG and Alexa Fluor 546-conjugated anti-rabbit IgG served as secondary antibodies. Nuclei were stained by 4′,6-diamidino-2-phenylindole (DAPI). Zeiss Axio Imager Z1 or Zeiss LSM 880 microscopes (Carl Zeiss AG, Oberkochen, Germany) were used for visualization.

## Data Availability

No datasets were generated or analysed during the current study.

## Appendix A

Case 2. A 62-year-old woman presented with a self-detected tumour in her right breast. Clinical breast examination showed a hard tumour in the upper-outer quadrant, clinically malignant (Figure A1).

Case 3. A 77-year-old woman presented with a self-detected tumour in her left breast. Clinical breast examination showed a hard tumour in the upper-outer quadrant, clinically malignant. Mammographic, ultrasound and histopathologic images are shown (Figure A2A); also for a synchronous nonpalpable tumour in the right breast (Figure A2B).
